# “More than just giving them a piece of paper”: Interviews with Primary Care on Social Needs Referrals to Community-Based Organizations

**DOI:** 10.1007/s11606-022-07531-3

**Published:** 2022-04-14

**Authors:** Laura B. Beidler, Na’amah Razon, Hannah Lang, Taressa K. Fraze

**Affiliations:** 1 The Dartmouth Institute for Health Policy and Clinical Practice, Geisel School of Medicine, Dartmouth College, Hanover, USA; 2 Family and Community Medicine, University of California, Davis, Davis, USA; 3 Philip R. Lee Institute for Health Policy Studies, University of California, San Francisco, San Francisco, USA; 4 Dartmouth College, Hanover, USA; 5 Family and Community Medicine, University of California, San Francisco, 3333 California Street, Suite 465, San Francisco, CA 94118 USA; 6 Healthforce Center, University of California, San Francisco, San Francisco, USA

**Keywords:** social determinants of health, primary care, clinicians, social risks, referrals

## Abstract

**Background:**

Primary care practices are responding to calls to incorporate patients’ social risk factors, such as housing, food, and economic insecurity, into clinical care. Healthcare likely relies on the expertise and resources of community-based organizations to improve patients’ social conditions, yet little is known about the referral process.

**Objective:**

To characterize referrals to community-based organizations by primary care practices.

**Design:**

Qualitative study using semi-structured interviews with healthcare administrators responsible for social care efforts in their organization.

**Participants:**

Administrators at 50 diverse US healthcare organizations with efforts to address patients’ social risks.

**Main Measures:**

Approaches used in primary care to implement social needs referral to community-based organizations.

**Results:**

Interviewed administrators reported that social needs referrals were an essential element in their social care activities. Administrators described the ideal referral programs as placing limited burden on care teams, providing patients with customized referrals, and facilitating closed-loop referrals. We identified three key challenges organizations experience when trying to implement the ideal referrals program: (1) developing and maintaining resources lists; (2) aligning referrals with patient needs; and (3) measuring the efficacy of referrals. Collectively, these challenges led to organizations relying on staff to manually develop and update resource lists and, in most cases, provide patients with generic referrals. Administrators not only hoped that referral platforms may help overcome some of these barriers, but also reported implementation challenges with platforms including inconsistent buy-in and use across staff; integration with electronic health records; management and prioritization of resources; and alignment with other organizations in their market.

**Conclusion and Relevance:**

Referrals to community-based organizations were used in primary care to improve patients’ social conditions, but despite strong motivations, interviewees reported challenges providing tailored and up-to-date information to patients.

**Supplementary Information:**

The online version contains supplementary material available at 10.1007/s11606-022-07531-3.

## INTRODUCTION

Responding to compelling evidence that links social risks—such as food, housing, transportation, or economic insecurity—to healthcare outcomes, primary care practices are considering how to improve patients’ social conditions (i.e., social care).^1–10^ Several forces have spurred the momentum to act on evidence linking social risks and healthcare outcomes including the ongoing shift towards value-based care in the Affordable Care Act and beyond, campaigns advanced by provider organizations such as the American Academy of Family Physicians, and influential reports by the National Academies of Sciences, Engineering, and Medicine and others.^11,12^ As a result, two-thirds of primary care practices report screening patients for at least one social risk factor (social conditions which are health risks).^13^ Information on patients’ social risk factors can be used by primary care clinicians to gain a deeper understanding of their patients’ lives, to adjust patient’s care plan (e.g., changes to medications or follow-up schedule), and to improve social conditions.^5,6,8,11^ For many primary care practices, the next step after social risk screening is likely providing patients with identified social needs (e.g., a social risk factor the patient experiences) with a referral to community-based organizations (CBOs).^4,5,11,13–23^

Social needs referrals are likely appealing to primary care settings because referrals capitalize on the expertise of CBOs and local resources rather than requiring healthcare organizations to independently develop social needs assistance programs such as housing or job placement support. Thus, primary care practices may use social needs referrals because they are a relatively low intensity social care intervention. Yet, primary care practices likely still face significant challenges providing social needs referrals due to workflow changes, staffing needed, and technology to support implementation.^14^

Despite evidence that primary care practices are screening patients for social risk factors,^6,24^ little is known about *how* they act to resolve patients’ social needs once identified. To address this gap, we interviewed healthcare administrators to explore their motivations, processes, and challenges associated with implementing social needs referrals. These findings can guide primary care practices as they develop and implement social needs referrals. Additionally, insights can inform policymakers and payers as they consider incentivizing social care as part of their effort to improve healthcare outcomes while managing costs.

## METHODS

We conducted semi-structured interviews with administrators at healthcare organizations between April 19 and July 26, 2019, to understand how social care activities are implemented in primary care. This study was approved by the Committee on the Protection of Human Subjects at Dartmouth College.

### Sample and Outreach

We used three strategies to identify healthcare organizations with current social care activities. First, we randomly sampled systems and primary care practices that reported conducting social risk screening (i.e., food, housing, transportation, utilities, and interpersonal violence) in the National Survey of Healthcare Organizations and Systems (NSHOS).^25,26^ Fielded from June 2017 to August 2018, NSHOS is a suite of nationally representative surveys that included a system-level survey (*N*=325, response rate=57%) and a primary care practice-level survey (*N*=2190; response rate=44%).^6,25–30^ Second, to ensure we sampled organizations that were meaningfully delivering social care, we identified primary care practices and health systems with publicly available information online about social care.

Third, we interviewed organizations participating in the Centers for Medicare and Medicaid Services’ (CMS) Accountable Health Communities (AHC) model.^31,32^ The AHC model, which began in 2017 (screening began in 2018), tests the impact of social care activities for Medicare and Medicaid beneficiaries within primary care, emergency department, behavioral health, and labor and delivery settings. The AHC model included 2 tracks: alignment and assistance. AHCs are required to screen patients for social risks in clinical settings. If a beneficiary screened positive for social risks, they are given a referral. High-risk beneficiaries were also eligible for assistance accessing resources (in the assistance track, beneficiaries were randomly assigned to receive assistance). Alignment AHCs were required to engaged community resources via an advisory committee.^31–34^

At each organization, we emailed an identified leader and asked them to connect us with the individual best suited to discuss their social care activities. Individuals identified by organizational leaders varied in formal titles and roles (Appendix Table 1), but all were responsible for overseeing programming. Interviewees were typically executive leadership (i.e., Chief Executive Officer, Chief Clinical Officer) or program management (i.e., Program Manager, Community Relations) who assumed the day-to-day management of social care activities (Appendix Table 1, Table 4, Table 5).

For the samples identified via NSHOS and Internet searches, we contacted a total of 64 organizations (Appendix Figure 1 details our outreach). Twenty-nine organizations responded and agreed to participate (Appendix Tables 2 and 3 summarize invited organizations). At four organizations, we conducted a second interview to gather additional information about social care activities (Appendix Table 4).

For the AHC sample, we contacted bridge organizations (which are centralized program management offices required in the AHC model) at all AHCs (*N*=31) participating in the model as of April 2019.^32^ We interviewed administrators at 22 (of 31) AHCs (Appendix Table 5). At 8 AHCs, we conducted secondary interviews with additional staff members (13 interviews). Two organizations interviewed in the non-AHC sample also participated in the AHC model as clinical sites. We asked these interviewees about activities within and outside of the AHC model and they are counted in both samples, but not twice in the overall number of interviews. The formal evaluation report sponsored by CMS provides detailed information on how AHCs implemented model requirements.^31^

We conducted outreach in waves to ensure diversity in respondents and continued interviews until we reached a point of saturation.^35^

### Data Collection and Analysis

Trained qualitative interviewers (T.F., a PhD-level health services researcher with advanced training in qualitative research; and L.B., an MPH-level researcher with expertise in healthcare delivery and qualitative methods) conducted the interviews and lead analyses. We followed a semi-structured interview guide that focused on organizational social care efforts including social risk screening, social needs referrals, case management assistance, and partnerships with CBOs (Appendix Table 6). Interviews were conducted via telephone and lasted approximately 1 h. After obtaining consent, interviews were recorded and then professionally transcribed.

We began coding during data collection. First, we coded all transcripts using NVivo.^36^ The initial coding was broad and used a codebook that was developed prior to coding and aligned with the interview guide domains. The initial coding was conducted by the lead author (L.B.) and a trained research assistant (H.L.). Intercoder reliability was established by having both coders and the senior author (T.F.) iteratively double-code transcripts and discuss discrepancies (Appendix Figure 2).^37^

We analyzed data on the implementation of social needs referrals in primary care. We conducted intermediate coding where two individuals (L.B. and H.L.) iteratively sub-coded data on social needs referrals that was then reviewed by the senior author (T.F.) with any discrepancies resolved via discussion. To organize and support analyses, we (L.B., H.L., and T.F.) developed an analytic memo that described all observed themes including how each organization fit within each theme. We used a matrix coding approach to justify the inclusion or exclusion of organizations within theme.^38^

We analyzed transcripts from each sample separately because we expected differences in approaches given the rigorous requirements of the AHC model. *Observed themes were consistent across samples*. Given this consistency, we report findings pooled across samples. We also examined if approaches varied: (1) by organizational characteristics (e.g., size, ownership, geography) or (2) by the level of the respondent (e.g., administrator in a health system vs. practice) but we did not observe any systematic differences.

## RESULTS

We conducted 66 interviews at 50 healthcare organizations. Interviewed organizations included 12 healthcare systems, 1 contracting organization similar to a healthcare system, 9 multi-site multispecialty practices, 6 primary care practices, and 22 AHC bridge organizations (16 alignment and 6 assistance tracks) (Appendix Tables 4 and 5). To protect confidentiality of AHC participants, we are unable to provide detailed information on the organizational characteristics; detail on all AHC model participants can be accessed via CMS’ evaluation year 1 report.^31^

All interviewed healthcare organizations (*N*=50) relied on social needs referrals to CBOs as a key component of their effort to improve patients’ social conditions because it allowed them to capitalize on external expertise while limiting the impact of social care efforts on their own organizations.

Then it’s referring people to the food banks because I don’t have capacity to run, nor should I have a food bank in every one of my clinics. That would be redundant for what’s in the community. – Health system

### Characteristics of Ideal Social Needs Referral Programs:

Administrators described three characteristics of an ideal referral program. First, social needs referrals should be low intensity and minimize the burden of providing referrals on care teams such that anyone could offer referrals to patients. Second, administrators emphasized that referrals should be tailored to each patient (e.g., referral to a food bank located along the patients’ bus route, that was open when the patient was available, or that offered culturally appropriate food). Third, most administrators wanted closed loop referral systems (bidirectional information sharing with CBOs) to ensure the patient accessed the resource, to determine if the CBO successfully assisted the patient, and to assess the impact of referrals on health care outcomes.

### Challenges with Implementing Referrals:

While the implementation processes, such as workforce used to implement social needs referrals, the types of patients who were eligible for referrals, and the degree of integration of referrals within clinical workflows, varied by interviewed organizations, we identified cross-cutting implementation challenges. Administrators reported three main challenges to implementing the ideal social needs referrals: developing and maintaining resource lists, aligning referrals with patients, and measuring the impact of referrals.

#### Challenge 1: Develop and Maintain Resource Lists

Developing and maintaining referral lists was time-consuming. Most (19 from the non-AHC sample; 9 from the AHC sample) relied on their own staff to identify and document key requirements for each resource, regularly update lists, and integrate feedback from colleagues and patients (Table 1): “We try to update it every six months. We don’t always get around to doing it at six months. Then we try and tell the staff, ‘if you know of any new resources, add it in the database’”- AHC.

**Table 1:** Developing and Managing Referral Lists Required Substantial Staff Time

	Organizations emphasized that the process of developing and maintaining referral lists was substantially more challenging and iterative than anticipated.	
Identify	Staff identified and compiled resources in their community. Healthcare staff called CBOs to determine services, eligibility requirements, and contact information	“Literally doing web searches for resources in the area and asking word of mouth. […] But then we also tasked our clinics to help us out. We gave them a very early draft of our resource inventories and asked them to double check things to see if we were missing anything or if they knew of resources that maybe we weren’t able to find.” - AHC
Confirm	Due to the dynamic nature of CBOs, staff regularly called to confirm information was still accurate. This was either done on a regular schedule (e.g., once every 6 months) or ad hoc	“[I]t’s never static, so things constantly change, but it’s not like it’s total chaos.” - Health System
Incorporate feedback	Healthcare organizations used feedback from staff and patients to refine referral lists (e.g., adding new resources or updating information on existing resources)	“‘Hey patient called resource X, the phone number doesn’t work. Here’s the correct one.’ We’re constantly relying on feedback from our clinics, and from the patients.” - AHC

Notes: *CBOs* community-based organizations, *AHC* accountable health communities

Administrators reported looking for technological solutions that would limit the amount of time their staff spent maintaining resource lists.

#### Challenge 2: Align Referrals with Individual Patients

Tailored referrals required significant knowledge of the patients’ needs that can be paired with a deep understanding of community resources and, of course, staff available to maintain and apply such knowledge (Table 2).

**Table 2:** While Tailored Referrals Were the Goal, Generic Referrals Were Less Labor Intensive for Care Teams to Implement

	Tailored referrals Incorporated patient’s specific needs.	Pre-existing lists were modified or prioritized.	Generic referrals Information was limited by need and/or location.
Staff	Pair patients with specific CBOs.	Prioritize pre-existing lists	Give patients pre-existing lists
	“She might put a note in the computer. ‘Referral made to Salvation Army. Spoke to whoever there. Supposed to meet patient at 2:00 PM.’ […] She usually will either call later today or first thing in the morning and just say, ‘Hey, just following up with you. Were you able to connect with Erin at 2:00?’” – Practice	“It’s actually not tailored specifically, but the screeners are really good and have gone to most of the community partners who are helping us with the food piece” – AHC	“Typically, what happens is if they recognize a need then you can actually print that screen with all of the information on it. They’ll print that out for the patient and give it to the patient.” – Practice
Patients	Guided and coached by staff in selecting and contacting CBOs.	Guided in selecting CBOs but expected to address needs on their own.	Expected to independently select CBOs from a generic list.
	“The navigator helps them to, not just gives them a bunch of resources, but gives them the resources and follows up with them and makes the first contact and closes the loop.” – System	“Then I call the patient and give them the information, tell them that I’ve given them the contact information as well. And that I encourage them to call. And if there’s any problem to contact me.” – Practice	“It’s essentially connecting them and giving them the information and then it’s up to the patient to contact those organizations and move the steps forward.” – Practice
Technology	Platforms used to select CBOS and to communicate with CBOs and patients.	Referral platforms are used to generate referral lists with limited tailoring.	Referrals were not autogenerated or customized.
	“They can print the program page right there and give them the information printed, email it to them, or we also have promotional cards so that patients can search the system on their own. It’s a public website, […] anyone in the community can go on and just find the help that they need on their own.” – Health system	“We prioritized [CBOs] one to five. One would be, should always show up. Two would be, should show up if there’s space in the ... We set a limit to the number of pages that could show up for a community resource summary. – AHC	“We use a paper-based method, and again, that’s because logistically that is a much quicker and simpler way for our staff to do it.” – Health system

Notes: *CBOs* community-based organizations, *AHC* accountable health communities

They have to […]select the resources, so they’re using a little bit of their own knowledge of the patient, what they can glean from the screening tool, what they’ve gotten about the demographic and what they know about the resources. -AHC

Given these pragmatic challenges, most organizations instead provided more generic information to patients based on the type of need (e.g., housing, food) or geographic location (e.g., resources by ZIP Code). This allowed administrators to scale activities because anyone in the clinic could give a patient a pre-printed list of resources without disrupting (or adding to) clinical workflows. When social needs referrals were more generic, it became the patients’ responsibility to identify the CBO that might best fit their needs. Some organizations helped patients select ideal CBOs:

They will specifically say to the patient, ‘Hey, here’s this list on all this food stuff. These three are the ones that are going to address your need and when you go Tonya is the girl that works at the front desk. She always wears a flower behind her ear.’– AHC

These challenges were amplified for AHCs because they were required to screen and refer a high volume of patients which resulted in most AHCs using pre-printed referrals.

#### Challenge 3: Measure the Impact of Social Needs Referrals

Administrators hoped referrals to CBOs were effective at improving patients’ social conditions, but administrators also expressed uncertainty about the efficacy of referrals. Since patients were neither involved in the development of referrals programs nor formally engaged throughout the process for feedback, administrators were unsure if referrals offered value to patients. For example, administrators were unsure if patients accessed CBOs, if patients had prior knowledge of the resource, or if their needs were fully addressed. Administrators thought that closed-loop referrals, where healthcare organizations can track if patients were able to access a resource and resolve their social needs, were necessary to assess the impact of referral programs. Many relied of care teams to manually “close the loop” as part of their effort to measure the impact of referrals on patient outcomes:

Or they did make the connection, and so then we know the next time we can reassess that and is that still a determinant? Are they still struggling with that, or is that getting better for them? We felt like we can link them with a resource, that if we didn’t add that follow up piece to it, it wasn’t really closing that loop. – Practice

### Potential for Referral Platforms to Mitigate Challenges:

Some organizations (6 from the non-AHC sample; 12 from the AHC sample) used or were planning to use referral platforms with the hope that by leveraging technology, their processes would be more efficient with fewer staffing resources needed to manage and track referrals. While referral platforms offered advantages, organizations also faced implementation challenges including (1) buy-in and consistent use by care teams and CBOs; (2) platform and electronic health record (EHR) integration; (3) management and prioritization of resources; and (4) alignment with other organizations in their market (Table 3). Administrators reported that these barriers prevented them from using referral platforms as envisioned. At the time of the interviews, referral platforms alone were not enough to overcome challenges associated with maintaining resource lists, tailoring referrals, and tracking the impact of referrals.

**Table 3:** Implementing Social Needs Referral Platforms Was More Challenging than Expected

Expectation	Reality	Challenges
Platform will be used by all clinical staff.	Only staff providing referrals use the platform.	• Staff are unaware of or uncomfortable using platform. • Referrals are often seen as the work of care management. • Staff may not have time during a clinical visit to use the platform.
“I suspect what will happen is that [referral platform] will be used by the front desk and the MA in the practice, and so by siphoning off those maybe lower risk patients who you can just use Aunt Bertha to get them to the right resource, that will not clog up the care manager workload.” – Health system	“Our original idea was to allow the data system to generate a tailored community referral summary by having the clinician, or the medical assistant check off the individual resources that they wanted to refer a patient to. But that very quickly became a very tedious task that was too much to ask of the clinics.” – AHC	
Integrate platform into existing technology.	Platforms and EHRs may not integrate easily.	• Staff have limited capacity to assume new responsibilities. • Referral platforms do not easily integrate into EHRs, requiring staff to toggle between programs.
“So, that is sort of a future functionality that we’re talking to [referral platform] about, is how do we take the information we collect from the screening tool and match it to the eligibility criteria that’s listed in the resources to create an even more refined list.” – AHC	“We have this integration whereby the screening results are sent in real time and the patient’s demographic information to [platform]. The clinical assistant does need to click into [platform]. It’s a same sign-on, not single sign-on.” – AHC	
Resource list will be managed by the vendor.	Platforms still require time to set-up and update.	• Informal CBOs may not be included in platforms (e.g., churches). • Updates in the referral platform may be cumbersome.
“A challenge across keeping some sort of a community-based resource list is upkeep of it and who owns it, so this, we know will be something that’s kept updated by [referral platform]” – Health system	“They’re constantly changing and so you need to have the ability to make sure that if you have a database of resources like that, that it’s kept up to date. I don’t think there’s a great system for that yet.” - Health system	
CBOs will use platforms for closed loop referrals.	Closed loop referrals require workflow changes.	• CBOs have existing systems for managing patients • CBOS may not want to adopt the referral platform. • Health care organizations and CBOs may not have the staffing capacity.
“Once [referral platform] is up, we will have far more capability of not just sending a referral but tracking the referral. Because as you know, right now for most of us in the world, when we give people information or even if we set up a referral, we don’t know what happens.” – Health system	“We tried to push the closing the loop piece right away, and what we learned is that we really need to get people in the system, used to it, navigating it, make sure people feel comfortable that everything’s accurate in the system. And then we can start really partnering with some of our key [CBOs], to start pilot testing...” - Health system	
Platform will serve as a community resource.	Multiple organizations may launch a platform.	• Challenging for health care organizations to share a platform across a market • CBOs may not be able to effectively partner with multiple platforms and health care organizations.
“The way the interface is being developed, the patients themselves will also be able to interact with it.” – Health system	“And so the risk with a potential separate system was that we would end up without a community centered solution, but disparate systems, which would create more chaos in the community organizations” - Health system	

Notes: *CBOs* community-based organizations, *AHC* accountable health communities, *EHR* electronic health record

## DISCUSSION

Social needs referrals may be pursued by healthcare organizations because referrals are viewed as the most manageable approach to delivering social care. Regardless of the intensity of their approach to social needs referrals—from generic pre-printed lists to highly personalized lists curated by staff—administrators felt referrals required substantial staffing investments (and, therefore, social care activities were costly).^39^ Developing, implementing, aligning, and evaluating social needs referrals represented significant challenges for healthcare organizations.

The use of social needs referrals within primary care to improve patients’ social conditions is not surprising as it allows healthcare to capitalize on the expertise and resources of others. Administrators believed that social needs referrals were easier to implement because there was less disruption to clinical workflows than directly assisting patients (i.e., delivering food to patients). Yet, administrators emphasized that social needs referrals were more challenging than expected. Administrators struggled to balance the use of pragmatic generic social needs referrals with the personalized, tailored referrals that they felt were more effective. As policymakers consider incentivizing social needs referrals, further research to determine what makes an effective referral (e.g., degree of tailoring needed) and defining which referral elements are essential will aid implementation.

Administrators expressed noteworthy concerns around the efficacy of social needs referrals.^40^ There is some evidence that social needs referrals, especially within pediatric settings, can improve care outcomes.^23,41,42^ Yet, significant gaps in the literature remain. Our study highlights concerns around patients’ baseline knowledge of CBOs (e.g., if referrals offer value), how often patients access resources included in referrals, and if those resources improve social conditions and clinical outcomes in the long-term. Healthcare organizations considering how they can improve patients’ social conditions could benefit from guidance on how to effectively structure and implement social needs referrals, particularly around the inclusion of patients’ perspectives.^43^ Greater guidance from and engagement with patients will help healthcare organizations better understand the efficacy of social needs referrals.

Technology-based solutions to social needs referrals, such as platforms NowPow or Aunt Bertha, have garnered significant interest and investment across the healthcare sector.^39,44–46^ Interviewees reported high hopes that platforms might ease implementation of social needs referrals, increase value for patients through more tailored referrals, and demonstrate the efficacy of their efforts by tracking CBO resource utilization. At least in the relatively early stages of using referral platforms among interviewees, the reality was not quite as hoped. This finding aligns with challenges reported in other studies, particularly the challenge of recruiting CBOs to actively use the platform.^22,39,45,47^ Referral platforms alone cannot resolve challenges around the hard work of developing meaningful cross-sector collaboration with CBOs and achieving buy-in across clinical teams.

Ideally, referral platforms can be used as a tool to facilitate community-wide engagement around improving population health. Yet, few of the interviewed healthcare organizations designed social needs referrals programs with the input of either patients or CBOs. Social needs referrals programs were designed to deliver social care while mitigating the impact on clinical care activities. While pragmatic and understandable, this approach, from the beginning, limits engagement of local CBOs whose expertise and buy-in is needed to achieve long-term improvements in community health.^48,49^ Policymakers considering how to incentivize social care in clinical settings could require more meaningful engagement between healthcare and CBOs such as by requiring cross-sector leadership for social care activities, reimbursement to CBOs for services, or requiring CBOs participate in and receive a portion of shared savings in value-based contracts.

Our study has key limitations. First, our data were largely from the perspective of administrators who were responsible for overseeing social care activities which may not represent the views of clinicians or others within the organization. Qualitative interviews are not meant to be generalized, rather interviews provide context and background on how healthcare organizations provide social needs referrals. Finally, these findings are from organizations that were committed to delivering social care and may not represent the views of organizations not already delivering social care. As a result, the challenges we identified may be even greater for organizations starting to deliver social care.

There is growing interest among healthcare delivery systems, payers, and policymakers on how to leverage the expertise and capacity of local resources to improve patients’ health. Among our interviewees, social needs referrals were viewed as the logical approach to improving patients’ social needs. Yet, there were also concerns as to whether healthcare organizations could deliver social needs referrals which are relevant and valuable to patients while also successfully balancing constraints around staffing and capacity. As heathcare organizations increasingly invest in social care, they could use guidance from policymakers and research on how to effectively structure social needs referrals.

## Supplementary Information

ESM 1 (DOCX 78 kb)

## Declarations

The Accountable Health Communities Model was funded by the Centers for Medicare & Medicaid Services. The statements presented in this paper do not necessarily reflect the views or policies of the Centers for Medicare & Medicaid Services. The authors assume responsibility for the accuracy and completeness of the information presented in this paper.
