# Supplementary material for: “More than just giving them a piece of paper”: Interviews with Primary Care on Social Needs Referrals to Community-Based Organizations
Source: J Gen Intern Med. 2022 Apr 14;37(16):4160–7. doi: 10.1007/s11606-022-07531-3 (PMC9708990; doi:10.1007/s11606-022-07531-3)
Supplement: Supplementary file 1 — (DOCX 78 kb) [file 11606_2022_7531_MOESM1_ESM.docx]

Appendix

Figure 1: Outreach overview

**Sample**

**Contacted**

**Interviewed**

**Secondary Interviews**

*Two interviewed organizations in the non-AHC sample also participated in the AHC model as clinical delivery sites. We asked these interviewees about both activities within and outside of the AHC model.

Appendix Figure 2: Analytic Approach

Table 1: Interviewee Categories

| **Interviewee Categories** | **Description** | **Examples** |
| --- | --- | --- |
| Executive Leadership | Individuals primarily responsible for overseeing the operations of the entire organization | Chief Executive Officer, Chief Clinical Officer |
| Program Management | Individuals who oversee specific departments or services | Program Manager, Community Relations Manager, Eligibility Supervisor |
| Case Management Staff | Individuals who worked within case management teams and who focused primarily on case management activities | Nurse Care Manager, Community Health Worker, Social Worker, Navigator |
| Practicing Clinician | Individuals whose primary role was the provision of medical care | Physician |

Appendix Table 2: Characteristics of all invited organizations in national sample

| **Site** | **Organization type** | **Region** | **Safety net practice**** | **Primary care only***** | **Practice**  **urbanicity***** | **Outreach Wave*** | **Participate in interview?** |
| --- | --- | --- | --- | --- | --- | --- | --- |
| 1 | Practice | Midwest | No | Yes | Urban | 1 | Yes |
| 2 | Health system | Northeast |  |  |  | 1 | Yes |
| 3 | Practice | West | Yes | Yes | Multiple | 1 | Yes |
| 5 | Health system | West |  |  |  | 1 | Yes |
| 6 | Practice | South | Yes | Yes | Rural | 1 | Yes |
| 7 | Health system | Northeast |  |  |  | 1 | Yes |
| 9 | Practice | West | Yes | No | Suburban | 1 | Yes |
| 11 | Health system | Northeast |  |  |  | 1 | Yes |
| 29 | Health system | South |  |  |  | 1 | Yes |
| 31 | Health system | Northeast |  |  |  | 1 | No |
| 32 | Health system | Midwest |  |  |  | 1 | No |
| 4 | Health system | West |  |  |  | 2 | Yes |
| 8 | Health system | Northeast |  |  |  | 2 | Yes |
| 10 | Practice | West | Yes | No | Urban | 2 | Yes |
| 12 | Health system | Northeast |  |  |  | 2 | Yes |
| 13 | Practice | Northeast | No | Yes | Rural | 2 | Yes |
| 15 | Practice | West | Yes | No | Suburban | 2 | Yes |
| 23 | Health system | Northeast |  |  |  | 2 | Yes |
| 30 | Practice | Northeast | Yes | Yes | Urban | 2 | No |
| 33 | Health system | West |  |  |  | 2 | No |
| 34 | Health system | South |  |  |  | 2 | No |
| 35 | Health system | West |  |  |  | 2 | No |
| 36 | Health system | Midwest |  |  |  | 2 | No |
| 37 | Health system | South |  |  |  | 2 | No |
| 14 | Practice | South | Yes | Yes | Suburban | 3 | Yes |
| 19 | Health system | Midwest |  |  |  | 3 | Yes |
| 21 | Practice | West | Yes | Yes | Suburban | 3 | Yes |
| 22 | Health system | Northeast |  |  |  | 3 | Yes |
| 25 | Health system | South |  |  |  | 3 | Yes |
| 38 | Health system | Midwest |  |  |  | 3 | No |
| 39 | Health system | South |  |  |  | 3 | No |
| 40 | Health system | Northeast |  |  |  | 3 | No |
| 41 | Health system | South |  |  |  | 3 | No |
| 16 | Practice | West | No | Yes | Urban | 4 | Yes |
| 17 | Practice | Midwest | Yes | Yes | Rural | 4 | Yes |
| 18 | Practice | West | Yes | Yes | Rural | 4 | Yes |
| 20 | Health system | Northeast |  |  |  | 4 | Yes |
| 24 | Health system | Midwest |  |  |  | 4 | Yes |
| 42 | Practice | Northeast | Yes | Yes | Urban | 4 | No |
| 43 | Practice | South | Yes | Yes | Rural | 4 | No |
| 44 | Practice | West | No | No | Urban | 4 | No |
| 45 | Practice | South | Yes | No | Multiple | 4 | No |
| 46 | Practice | South | Yes | Yes | Multiple | 4 | No |
| 47 | Health system | South |  |  |  | 4 | No |
| 48 | Health system | Midwest |  |  |  | 4 | No |
| 49 | Health system | South |  |  |  | 4 | No |
| 50 | Health system | West |  |  |  | 4 | No |
| 51 | Health system | West |  |  |  | 4 | No |
| 26 | Practice | South | Yes | Yes | Urban | 5 | Yes |
| 27 | Practice | Midwest | Yes | Yes | Urban | 5 | Yes |
| 52 | Practice | South | No | Yes | Suburban | 5 | No |
| 53 | Practice | West | Yes | Yes | Multiple | 5 | No |
| 54 | Practice | Northeast | No | Yes | Urban | 5 | No |
| 55 | Practice | Northeast | No | Yes | Urban | 5 | No |
| 56 | Practice | South | Yes | Yes | Suburban | 5 | No |
| 61 | Practice | Northeast | Yes | Yes | Suburban | 5 | No |
| 62 | Practice | West | No | Yes | Multiple | 5 | No |
| 28 | Practice | Northeast | No | Yes | Rural | 6 | Yes |
| 57 | Practice | Midwest | No | Yes | Suburban | 6 | No |
| 58 | Practice | South | No | Yes | Urban | 6 | No |
| 59 | Practice | West | No | Yes | Suburban | 6 | No |
| 60 | Practice | Midwest | No | Yes | Rural | 6 | No |
| 63 | Health system | South |  |  |  | 6 | No |
| 64 | Health system | West |  |  |  | 6 | No |

*Outreach was conducted in rolling waves with each wave being dependent upon the organization that agreed to participate from earlier waves. The number and type of organizations in each wave were selected to help provide a robust and diverse sample.

** We did not collect data on if a system could be designated as predominately safety net.

***Urbanicity and specialty mix are only reported for practice as most systems have both primary and specialty care, and span levels of urbanicity.

Appendix Table 3: Summary of participating and non-participating organizations in national sample

|  | **Participating organizations (n=29)** | **Non-participating organizations***  **(n=35)** |
| --- | --- | --- |
| **Organizational type** |  |  |
| Health system | 14 (48.3%) | 18 (51.4%) |
| Practice | 15 (51.7%) | 17 (48.6%) |
| **Region** |  |  |
| Midwest | 5 (17.2%) | 6 (17.1%) |
| Northeast | 10 (34.5%) | 7 (20.0%) |
| South | 5 (17.2%) | 13 (37.1%) |
| West | 9 (31.0%) | 9 (25.7%) |
| **Practice specialty mix**** |  |  |
| Primary care only | 12 (80.0%) | 15 (88.2%) |
| Multi-specialty | 3 (20.0%) | 2 (11.8%) |
| **Safety net practice***** |  |  |
| Yes | 11 (73.3%) | 8 (47.1%) |
| No | 4 (26.7%) | 9 (52.3%) |
| **Practice urbanicity**** |  |  |
| Urban | 5 (33.3%) | 6 (35.3%) |
| Suburban | 4 (26.7%) | 5 (29.4%) |
| Rural | 5 (33.3%) | 2 (11.8%) |
| Multiple | 1 (6.7%) | 4 (23.5%) |

*These organizations were contacted and asked to participate in this study, but they did not respond to outreach.

**Urbanicity and specialty mix are only reported for practice as we expect most systems to have both primary and specialty care and to span levels of urbanicity.

***We did not collect data on if a system included any safety net providers.

Table 4: National sample organizational characteristics

| **Site** | **Description** | **Composition** | **Interviews** | **Interviewee(s) Role*** | **Reason for second interview** |
| --- | --- | --- | --- | --- | --- |
| 1 | Urban family medicine clinic in the Midwest (10 to 20 providers) | Single primary care delivery site | 1 | Program Management |  |
| 2 | Health system in the Northeast | Hospital, primary care and specialty delivery sites | 1 | Program Management (2) |  |
| 3 | Coalition of community health centers in the West | Primary care clinics | 1 | Executive Leadership, Program Management |  |
| 4 | Health system in the West | Hospitals, primary care and specialty delivery sites | 1 | Executive Leadership |  |
| 5 | Health system in the West | Hospitals, primary care and specialty delivery sites | 1 | Executive Leadership |  |
| 6 | Rural FQHC in an area that covers two states in the South (1 to 10 providers) | Single primary care delivery site | 1 | Executive Leadership |  |
| 7 | Health system in the Northeast | Hospitals, primary care and specialty delivery sites | 1 | Executive Leadership, Program Management (2) |  |
| 8 | Rural healthcare system that includes hospitals in two states in the Northeast | Hospitals, primary care and specialty delivery sites | 2 | Executive Leadership, Program Management/Practicing Clinician | First interviewee suggested that the second interviewee may have additional insight as a practicing clinician involved in program implementation |
| 9 | Suburban FQHC with multiple clinical delivery sites in the West | Primary and specialty care delivery sites | 1 | Program Management |  |
| 10 | Urban FQHC with multiple locations in the West | Primary and specialty care delivery sites | 1 | Program Management |  |
| 11 | Accountable care organization in the Northeast | Hospitals, primary care and specialty delivery sites | 1 | Executive Leadership |  |
| 12 | Large health system in the Northeast that also manages its own health plan | Hospitals, primary care and specialty delivery sites; health plan | 2 | Executive Leadership, Case Management Staff | First interview discussed program goals across the system, secondary interview provided details on the case management process |
| 13 | Small rural practice in the Northeast | Single primary care delivery site | 1 | Program Management (2), Practicing Clinician |  |
| 14 | Large suburban FQHC in the South | Primary care delivery sites | 1 | Executive Leadership, Program Management, Case Management Staff |  |
| 15 | FQHC suburban in the West | Primary and specialty care delivery sites | 1 | Program Management |  |
| 16 | Urban practice in the West (less than 10 providers) | Single care delivery site | 1 | Case Management Staff |  |
| 17 | Rural community health center in the Midwest (20 to 40 providers) | Primary care delivery sites | 1 | Case Management Staff |  |
| 18 | Rural community health center in the West | Primary care delivery sites | 1 | Program Management |  |
| 19 | Health system in the Midwest | Hospitals, primary care and specialty delivery sites | 1 | Program Management (2) |  |
| 20 | Urban system in the Northeast | Hospitals, primary care and specialty delivery sites | 1 | Executive Leadership, Program Management |  |
| 21 | Suburban community health center in the West | Primary care delivery sites | 2 | Executive Leadership, Program Management | First interviewee suggested that second interviewee would be able to explain details to implementation of the programming within clinics |
| 22 | Health system in the Northeast | Hospitals, primary care and specialty delivery sites | 1 | Executive Leadership |  |
| 23 | Health system in the Northeast | Hospitals, primary care and specialty delivery sites | 2 | Program Management |  |
| 24 | Health system in the Midwest | Hospitals, primary care and specialty delivery sites | 1 | Executive Leadership, Program Management (2) | First interviewee provided an overview of broad strategic goals, secondary interviewee explained the details of program design and implementation |
| 25 | Health system in the South | Hospitals, primary care and specialty delivery sites | 1 | Program Management |  |
| 26 | Urban community health center in the South | Primary care delivery sites | 1 | Executive Leadership |  |
| 27 | Urban FQHC in the Midwest (20 to 50 providers) | Single primary care delivery | 1 | Executive Leadership |  |
| 28 | Rural independent practice in the Northeast (less than 10 providers) | Single primary care delivery site | 1 | Practicing Clinician |  |
| 29 | Urban academic health system in the South | Hospitals, primary care and specialty delivery sites | 1 | Executive Leadership, Program Management |  |

*The number in parentheses indicated if there were more than 1 individual from an interviewee category who participated in the interview

Table 5: AHC sample characteristics

| **Organization Number** | **Number of interviews** | **AHC Track** | **Interviewee(s) Role*** |
| --- | --- | --- | --- |
| 1 | 1 | Alignment | Program Management (4) |
| 2 | 3 | Alignment | Program Management, AHC staff (2) |
| 3 | 1 | Alignment | Program Management |
| 4 | 1 | Alignment | Executive Leadership, Program Management (3) |
| 5 | 1 | Alignment | Executive Leadership, Program Management |
| 6 | 1 | Assistance | Program Management (2) |
| 7 | 1 | Alignment | Executive Leadership, Program Management |
| 8 | 1 | Alignment | Program Management (2) |
| 9 | 1 | Alignment | Executive Leadership, Program Management (2) |
| 10 | 1 | Alignment | Executive Leadership (2), Program Management |
| 11 | 3 | Alignment | Program Management (3), AHC staff (2) |
| 12 | 1 | Alignment | Program Management |
| 13 | 2 | Alignment | Executive Leadership, Care management staff |
| 14 | 2 | Alignment | Program Management, Care Management staff |
| 15 | 2 | Assistance | Executive Leadership, Program Management, AHC staff |
| 16 | 4 | Assistance | Executive Leadership (3), Program Management (2), AHC staff (2) |
| 17 | 2 | Alignment | Program Management, AHC Staff |
| 18 | 1 | Assistance | Executive Leadership |
| 19 | 1 | Assistance | Program Management (2) |
| 20 | 1 | Assistance | Program Management, Clinician |
| 21 | 1 | Alignment | Program Management |
| 22 | 1 | Alignment | Executive Leadership, Program Management |

*The number in parentheses indicated if there were more than 1 individual from an interviewee category who participated in the interview(s)

Table 6: Interview Guide Domains

| **Domain** | **Probes** |
| --- | --- |
| Organizational Characteristics | - Organization size and structure - Interviewee role - Motivations for social needs work - Populations served - Participation in delivery reforms |
| Screening | - Which patients screened - Needs screened for - Screening workflows, methods, tools used - Staff involved with screening - Follow-up processes - Access to screening results - Frequency of screening - Reason for starting screening - Development of screening program - Engagement with clinicians - Buy-in from staff - Plans for scaling, changing screening program - Common needs patients have |
| Referrals | - Workflow - Staff involved - Tailoring to patients - Variation between locations, patients - Referral lists, referral platforms   - Development   - Maintenance   - Staff involved   - Tracking use of referrals - Role of clinicians - Buy-in from clinicians and patients - Follow-up processes - Closed loop referrals - Common challenges with referrals |
| Assistance | - Workflow - Types of assistance offered - Staff involved - Staff training - Variation between patients - Engagement with community organizations - Communication with   - Patients   - Clinicians   - Other staff - Centralized vs. decentralized programs - Tracking of patients/data collection - Common challenges - Reason for starting assistance work - Program development - Changes made to program |
| Need specific programming (e.g., food, housing, transportation) | - Internal programs - External programs - Funding - Services - Types of patients - Development processes |
| Interactions with community-based organizations (CBOs) | - Types of partners - Role of:   - Health care organization   - CBO - History of partnerships - Involvement of CBO in program development - Formalized or ad-hoc - Contractual relationships - Types of patients served - Data/records sharing |
| Overview/Reflection | - Challenges faced - Challenges solved - Overlap with care management - Organizational buy-in - Advice for other organizations - Organizational goals - Needed support (financial, resources, policy) |
